# Association Between Low Bone Mineral Density Risk Factors and Estrogen Receptor α Gene Polymorphisms in Japanese Female Athletes

**DOI:** 10.1089/whr.2020.0106

**Published:** 2021-01-11

**Authors:** Tetsuro Kobayashi, Inkwan Hwang

**Affiliations:** Faculty of Sports Science, Nippon Sport Science University, Yokohama, Japan.

**Keywords:** triad risk factors, gene polymorphisms, competitive sports, collegiate athletes

## Abstract

***Background:*** The relationship between bone metabolism-related gene polymorphisms and low bone mineral density (BMD) risk factors in female athletes is unclear. This study aimed at investigating whether the sensitivity of low BMD risk factors to BMD depends on estrogen receptor α (ERα) gene polymorphisms in Japanese female athletes.

***Materials and Methods:*** This study included 280 collegiate female athletes from 12 competitive sports (age, 19.2 ± 1.3 years). Data on sports participation, age at menarche, menstrual cycles, prior stress fractures, and prior eating disorders were obtained through a questionnaire-type survey. Sports types were classified into endurance, esthetic, aquatic, ball, and high load in consideration of exercise load characteristics. ERα gene *Pvu*II (rs2234693) and *Xba*I (rs9340799) polymorphisms were analyzed by TaqMan^®^ assay. The total body BMD was measured by dual-energy X-ray absorptiometry.

***Results:*** On multiple regression analysis, sports types, body mass index (BMI), menarche, and *Xba*I polymorphism remained robust independent predictors of BMD. However, prior stress fractures and menstrual cycles were excluded. In athletes carrying the XX+Xx genotype of *Xba*I polymorphism, sports types and BMI were associated with BMD. However, in athletes carrying the xx genotype of *Xba*I polymorphism, sports types, BMI, and menarche were associated with BMD. These results indicated that athletes carrying the xx genotype with delayed menarche had low BMD.

***Conclusions:*** In collegiate female athletes, participation in endurance, esthetic, and aquatic sports types and a low BMI are associated with low BMD. In addition, delayed menarche may negatively affect BMD in athletes carrying the xx type of ERα gene *Xba*I polymorphism.

## Introduction

Recently, low-energy availability, amenorrhea, and osteoporosis have been defined as the female athlete triad (the triad).^1^ In the triad, osteoporosis is characterized by low bone mineral density (BMD) and is a risk for serious injuries such as stress fractures for athletes. Therefore, risk factors for low BMD have been widely discussed. Previous studies have reported that low BMD risk factors include low body mass index (BMI),^2–5^ delayed menarche,^2–5^ menstrual abnormalities such as oligomenorrhea or amenorrhea,^2–6^ prior eating disorders,^2–4^ prior stress fractures,^2–5^ as well as participation in long-distance, aquatic, and esthetic types of sports.^4,7,8^ Moreover, a cumulative number of low BMD risk factors was associated with an increased risk of low BMD.^9^ Thus, low BMD risk factors in female athletes are being identified.

Previous studies have reported that BMD may be affected by differences in gene polymorphisms, and one of the bone metabolism-related gene polymorphisms is vitamin D receptor (VDR) gene polymorphism.^10^ The VDR gene has been targeted by research as a genetic determinant influencing bone status, because it regulates bone homeostasis through the vitamin D endocrine system. To date, there have been reports that VDR gene polymorphisms were associated with BMD^10–12^ or not associated with BMD,^13,14^ indicating inconsistent results. In addition, VDR gene polymorphisms have been reported to have environmental sensitivity to BMD.^15,16^ Hwang et al. applied this to female athletes to investigate whether the sensitivity of low BMD risk factors to bone response depends on VDR gene polymorphisms.^17,18^ As a result, they have indicated that menstrual abnormality was associated with decreased BMD in athletes carrying the Bb genotype of the VDR gene *Bsm*I polymorphism,^17^ and susceptibility to low BMD risk factors may be higher in athletes carrying the aa genotype of the VDR gene *Apa*I polymorphism than the AA+Aa genotype.^18^ However, they have stated that the individual differences in bone response cannot be determined by a single genetic polymorphism; thus, it is necessary to examine multiple genetic polymorphisms.

Currently, estrogen receptor (ER) gene polymorphisms are being studied alongside VDR gene polymorphisms as bone metabolism-related gene polymorphisms. Estrogens exert beneficial effects on the development and maintenance of the skeleton.^19^ Reports from population-based studies indicated that estrogen is necessary for the regulation of skeletal homeostasis not only in women but also in men.^20,21^ The skeletal effects of estrogen are mediated by its binding to specific ER localized at the cytosol and intranucleus.^19^ At least two main ER subtypes exist: ERα and ERβ. ERα gene consists of eight exons spanning 140 kb of the chromosome 6q25.1 locus,^22^ whereas ERβ gene consists of eight exons spanning ∼40 kb of the chromosome 14q23–24.1 locus.^23^ ERβ shows close structural homology with the ERα molecule, especially in the DNA-binding domain and, to a lesser extent, in the ligand-binding domain.^19^

Intron I polymorphisms (*Pvu*II and *Xba*I) upstream from the ERα gene were first associated with BMD variation in Japanese postmenopausal women. In this study, results of the statistical analyses indicated that the homozygote of the Px haplotype (PPxx) has significantly lower BMD values for both the lumbar spine and the total body than the other haplotypes. In contrast, no significant differences were found between haplotypes in terms of mean age, height, weight, number of years since menopause, and bone formation and resorption markers, suggesting that ERα gene polymorphisms may be a factor in post-menopause osteoporosis in Japanese women.^24^ Some studies have reported significant associations between ERα gene polymorphisms and BMD,^25–28^ whereas other studies reported no associations,^29–32^ indicating inconsistent results. Various suggestions have been made to account for these discordant findings, including genotyping error, ethnic or environmental differences among populations, differences in age and menopausal status, and study design.^33^ In addition, a recent study has reported that ERα gene polymorphisms have environmental sensitivity to BMD.^34^ However, participants in most previous studies were menopausal women, and no studies have focused on female athletes.

We hypothesized that differences in susceptibility to bone response of low BMD risk factors could be explained by ERα gene polymorphisms in female athletes. Therefore, this study aimed at investigating the effect of low BMD risk factors and ERα gene polymorphisms on BMD and whether the sensitivity of low BMD risk factors to BMD depends on ERα gene polymorphisms in Japanese female athletes.

## Materials and Methods

### Participants

In total, 280 female athletes from 12 competitive sports of a physical educational university (judo, *n* = 19; jumping/throwing of track and field, *n* = 10; weight lifting, *n* = 20; tennis, *n* = 29; soft tennis, *n* = 31; badminton, *n* = 10; hand ball, *n* = 32; long distance in track and field, *n* = 36; rhythmic gymnastics, *n* = 19; dancing, *n* = 22; water polo, *n* = 19; lifeguarding, *n* = 33) participated in this study. The participants competed in collegiate national championships and international events (*n* = 10, 3.6%). Physical characteristics of the study participants are shown in Table 1.

**Table 1.** Physical Characteristics of the Study Participants (*n* = 280)

	Mean ± SD
Age (years)	19.2 ± 1.3
Height (cm)	160.4 ± 4.7
Weight (kg)	56.5 ± 6.6
BMI (kg/m^2^)	22.0 ± 2.4
%Fat (%)	24.0 ± 4.0
Fat mass (kg)	13.7 ± 3.4
Fat free mass (kg)	40.4 ± 3.9
Experience of competition (years)	8.3 ± 4.2
Age at menarche (years)	12.9 ± 1.9
Total body BMD (g/cm^2^)	1.191 ± 0.096

BMD, bone mineral density; BMI, body mass index; SD, standard deviation.

All participants provided written informed consent for participation in this study, which was approved by the ethics review committee of the Nippon Sport Science University (019-G02). For participants aged <20 years, experiments were performed with the consent of the parents.

### Questionnaire

Data on sports participation, competition experience, age at menarche, menstrual cycles in the past year, prior stress fractures, and prior eating disorders were obtained through a questionnaire-type survey at the start of the experiment. For the menstrual cycles in the past year, participants chose one of the following: <25 days (polymenorrhea), 25–38 days (eumenorrhea), 39–89 days (oligomenorrhea), >90 days (amenorrhea), and fluctuated for >7 days (menstrual irregularity). Prior stress fracture was defined as a case where a doctor diagnosed stress fracture at least once, and prior eating disorder was described as a case where a doctor diagnosed anorexia nervosa or bulimia nervosa at least once.

### Dual-energy X-ray absorptiometry

Total body BMD, fat mass, and lean mass were measured by dual-energy X-ray absorptiometry (iDXA, GE Medical Systems Lunar, Madison, WI). All DXA data were acquired by a single radiological technician.

### Genotyping

In this study, 2 mL of saliva samples was collected by using DNA self-collection kit (Oragene^®^ DISCOVER, DNA Genotek, Canada). Collected saliva samples were incubated for 60 minutes by using a water incubator (SN-100SD, NISSIN) warmed up to 50°C. Thereafter, DNA was extracted by using a purified solution (PT-L2P-5) according to the Oragene DISCOVER's instructions. ERα gene *Pvu*II and *Xba*I polymorphism (rs2234693 and rs9340799, respectively) were genotyped by using the TaqMan SNP genotyping assay and the Real-Time PCR System (CFX96 Touch™ Real-Time PCR, Bio-Rad, Hercules, CA). TaqMan assays for genotype calls were analyzed by using CFX Manager Software version 2.1 (Bio-Rad).

### Classification of competition types

Considering the effect of the characteristics of sports types on BMD, 12 competitive sports were classified into five sports types, with reference to previous studies^4,7,8^: endurance type (long distance of track and field), esthetic type (rhythmic gymnastics and dancing), aquatic type (water polo and lifeguarding), ball type (tennis, soft tennis, badminton, and hand ball), and high-load type (judo, jumping/throwing in track and field, and weight lifting).

### Data analysis

BMI, age at menarche, menstrual cycles, prior eating disorders, and prior stress fractures were selected as risk assessment items for the triad in the American College of Sports Medicine (ACSM) consensus statement,^2^ and sports types and ERα gene polymorphisms were determined as assessment items for low BMD risk factors in the current study.

To perform multiple regression analysis with each assessment item as an independent variable and BMD as a dependent variable, each assessment item was defined as dummy variable 0 or 1 as follows: (1) BMI: The ideal BMI of 22 kg/m^2^ proposed by Tokunaga et al. was set for a reference value,^35^ with 1 as below the reference value and 0 as above the reference value. (2) Age at menarche: The average age at menarche in Japanese women (12.2 years) as shown by a previous study was set for a reference value,^36^ with 1 and 0 as above and below the reference value, respectively. (3) Menstrual cycles: Based on the menstrual cycle data in the past year obtained through a questionnaire-type survey at the start of the experiment, cases of oligomenorrhea, amenorrhea, or menstrual irregularity were defined as 1 and the others as 0. (4) Prior eating disorders: Cases with and without prior eating disorders were defined as 1 and 0, respectively. (5) Prior stress fractures: Cases with and without prior stress fractures were defined as 1 and 0, respectively. (6) Sports types: Participants participating in endurance type, esthetic type, or aquatic type were defined as 1, and participants participating in ball type or high-load type were defined as 0. (7) ERα gene polymorphisms: in *Pvu*II polymorphism, athletes carrying the PP or Pp genotype were defined as 1 and those with the pp genotype were defined as 0. In *Xba*I polymorphism, cases carrying the xx genotype were defined as 1 and those with the XX or Xx genotype as 0.

SPSS version 24.0 (IBM Corp., Armonk, NY) was used to perform all statistical analyses. The level of significance was set at *p* < 0.05. Pearson's chi-square test was performed to confirm the conformance of the observed genotype frequencies with the Hardy–Weinberg equilibrium distribution. Comparisons of the physical characteristics of participants between each sports type were performed by analysis of variance. If a significant effect was noted, a *post hoc* test using Bonferroni's multiple-comparison tests was completed to identify significant differences among the mean values. Pearson's correlation coefficient was used to investigate the relationship between each low BMD risk factor and BMD in all participants and each sports type. To investigate the effect of low BMD risk factors on BMD and whether the sensitivity of low BMD risk factors to BMD depends on ERα gene polymorphism, multiple regression analysis was performed by using the stepwise method with the assessment items as the independent variable (BMI, age at menarche, menstrual cycles, prior stress fractures, sports types, and ERα gene *Xba*I polymorphism) and BMD as the dependent variable in all participants and each *Xba*I polymorphism.

## Results

### Genotype frequency

Genotype frequencies of ERα gene *Pvu*II and *Xba*I polymorphisms in all participants and each competitive sport are shown in Table 2. Genotype frequencies of *Pvu*II (PP, 18%; Pp, 49%; pp, 33%) and *Xba*I (XX, 3%; Xx, 27%; xx,70%) polymorphisms were within the Hardy–Weinberg equilibrium (*p* = 1.000 and *p* = 0.410, respectively). In addition, because of low genotype frequencies of the PP and XX genotypes in the subsequent analysis, the PP+Pp genotype (67%) and the pp genotype (33%), and the XX+Xx genotype (30%) and the xx genotype (70%), were compared.

**Table 2.** Genotype Frequencies of *Pvu*II and *Xba*I Polymorphisms in the Estrogen Receptor α Gene in Each Sport

Sport	PP type	Pp type	PP+Pp type	pp type	HWE	XX type	Xx type	XX+Xx type	xx type	HWE
	*n* (%)	*n* (%)	*n* (%)	*n* (%)	*p*	*n* (%)	*n* (%)	*n* (%)	*n* (%)	*p*
Judo	3 (16)	10 (53)	15 (68)	6 (32)		1 (5)	4 (21)	5 (26)	14 (74)	
Jumping/throwing	1 (10)	5 (50)	6 (60)	4 (40)		0 (0)	1 (10)	1 (10)	9 (90)	
Weightlifting	3 (15)	12 (60)	15 (75)	5 (25)		0 (0)	12 (60)	12 (60)	8 (40)	
Tennis	4 (14)	19 (66)	23 (79)	6 (21)		0 (0)	7 (24)	7 (24)	22 (76)	
Soft tennis	4 (13)	17 (55)	21 (68)	10 (32)		3 (10)	5 (16)	8 (26)	23 (74)	
Badminton	0 (0)	7 (70)	7 (70)	3 (30)		0 (0)	2 (20)	2 (20)	8 (80)	
Handball	4 (13)	13 (41)	17 (53)	15 (47)		0 (0)	8 (25)	8 (25)	24 (75)	
Long-distance running	8 (22)	12 (33)	20 (56)	16 (44)		1 (3)	10 (28)	11 (31)	25 (69)	
Rhythmic gymnastics	1 (5)	11 (58)	12 (63)	7 (37)		0 (0)	5 (26)	5 (26)	14 (74)	
Dance	7 (32)	8 (36)	15 (68)	7 (32)		1 (5)	9 (41)	10 (45)	12 (55)	
Water polo	6 (32)	9 (47)	15 (79)	4 (21)		3 (16)	5 (26)	8 (42)	11 (58)	
Lifeguarding	10 (30)	14 (42)	24 (73)	9 (27)		1 (3)	7 (21)	8 (24)	25 (76)	
All participants	51 (18)	137 (49)	188 (67)	92 (33)	1.000	10 (3)	75 (27)	85 (30)	195 (70)	0.410

HWE, Hardy–Weinberg equilibrium; PP, *Pvu*II homozygous dominant; Pp, *Pvu*II heterozygous; pp, *Pvu*II homozygous recessive; XX, *Xba*I homozygous dominant; Xx, *Xba*I heterozygous; xx, *Xba*I homozygous recessive.

### Physical characteristics of each sports type

The physical characteristics of each sports type are shown in Table 3. For height, ball-type athletes showed a significantly higher value than high-load-type athletes (*p* < 0.05). Moreover, athletes in the high-load type had higher weight than athletes in other types (all *p* < 0.05). In contrast, athletes in the endurance type had lower weight than athletes in other types (all *p* < 0.05). BMI was similar to the statistical significance of weight. Athletes in the endurance type had lower %fat than athletes in other types (all *p* < 0.05). In terms of BMD, athletes in endurance (1.108 ± 0.067 g/cm^2^), esthetic (1.156 ± 0.083 g/cm^2^), and aquatic (1.149 ± 0.082 g/cm^2^) types had significantly lower values than athletes in the ball (1.217 ± 0.083 g/cm^2^) and high-load (1.275 ± 0.075 g/cm^2^) types, and athletes in the high-load type had higher values than athletes in the ball type (all *p* < 0.05).

**Table 3.** Physical Characteristics of Participants Belonging to Each Sports Type

	Endurance (*n* = 36)	Esthetic (*n* = 41)	Aquatic (*n* = 52)	Ball (*n* = 102)	High-load (*n* = 49)
Height (cm)	159.8 ± 3.7	160.2 ± 4.5	160.6 ± 4.5	161.4 ± 5.0^a^	158.7 ± 4.7^b^
Body weight (kg)	48.3 ± 3.6^a–d^	53.9 ± 5.7^a,b,d,e^	57.7 ± 5.3^a,c,e^	57.5 ± 4.7^a,c,e^	61.6 ± 7.1^b–e^
BMI (kg/m^2^)	18.9 ± 1.3^a–d^	21.0 ± 1.8^a,b,d,e^	22.4 ± 1.9^a,c,e^	22.1 ± 1.6^a,c,e^	24.4 ± 2.4^b–e^
%Fat (%)	19.6 ± 3.2^a–d^	23.8 ± 4.0^e^	25.1 ± 3.8^e^	24.6 ± 3.4^e^	24.8 ± 3.6^e^
Fat mass (kg)	9.4 ± 1.9^a–d^	13.0 ± 3.2^a,e^	14.6 ± 3.2^e^	14.2 ± 2.6^e^	15.5 ± 3.7^c,e^
Fat free mass (kg)	36.9 ± 2.6^a,b,d^	38.6 ± 3.3^a,b,d^	40.8 ± 3.4^a,c,e^	40.8 ± 3.3^a,c,e^	43.5 ± 4.0^a,c–e^
Total body BMD (g/cm^2^)	1.108 ± 0.067^a,b^	1.156 ± 0.083^a,b^	1.149 ± 0.082^a,b^	1.217 ± 0.083^a,c–e^	1.275 ± 0.075^b–e^

Data are presented as mean ± SD.

^a^ *p* < 0.05 versus high-load type (judo, jumping/throwing, and weightlifting).

^b^ *p* < 0.05 versus ball type (tennis, soft tennis, badminton, and handball).

^c^ *p* < 0.05 versus esthetic type (rhythmic gymnastics and dance).

^d^ *p* < 0.05 versus aquatic type (water polo and lifeguarding).

^e^ *p* < 0.05 versus endurance type (long-distance).

### Relationship between low BMD risk factor assessment items and BMD

In all participants, a significant negative correlation was observed between sports types (*r* = −0.499), BMI (*r* = −0.397), age at menarche (*r* = −0.236), prior stress fractures (*r* = −0.140), and menstrual cycles (*r* = −0.146), which were selected as low BMD risk factor assessment items, and BMD (all *p* < 0.05). In each sports type, the relationship between age at menarche and BMD in athletes in the aquatic type (*r* = −0.285), BMI and BMD in athletes in the ball type (*r* = −0.337), and prior stress fractures and BMD in athletes in the high-load type (*r* = −0.286) showed significantly negative correlations (all *p* < 0.05). In contrast, athletes in the endurance type showed no significant correlation between low BMD risk factor assessment items and BMD.

In addition, there tended to be a negative correlation between ERα gene *Xba*I polymorphism and BMD in all participants (*p* = 0.051), and a significant negative correlation was observed in athletes in the esthetic type (*p* < 0.05) (Table 4).

**Table 4.** Relationship Between Low Bone Mineral Density Risk Factors, Estrogen Receptor α Gene Polymorphisms, and Total Body Bone Mineral Density in Each Sports Type

	All participants (n = 280)		Endurance (*n* = 36)		Esthetic (*n* = 41)		Aquatic (*n* = 52)		Ball (*n* = 102)		High-load (*n* = 49)	
	*r*	*p*	*r*	*p*	*r*	*p*	*r*	*p*	*r*	*p*	*r*	*p*
Sports types	−0.499^*^	0.000										
BMI	−0.397^*^	0.000	0.134	0.435	−0.216	0.174	−0.251	0.073	−0.337^*^	0.001	−0.247	0.087
Age at menarche	−0.236^*^	0.000	−0.313	0.063	−0.242	0.128	−0.285^*^	0.041	−0.079	0.429	−0.106	0.469
Prior stress fractures	−0.140^*^	0.019	−0.197	0.249	0.194	0.222	0.098	0.491	−0.037	0.710	−0.286^*^	0.046
Menstrual cycles	−0.146^*^	0.015	0.215	0.302	0.044	0.783	0.184	0.191	−0.020	0.841	0.211	0.145
Prior eating disorders	0.058	0.345	−0.187	0.275	0.068	0.674	−0.103	0.469	−0.075	0.454	−0.269	0.062
ERα gene *Pvu*II polymorphism	0.050	0.401	0.277	0.103	0.238	0.135	−0.074	0.601	−0.023	0.820	−0.069	0.640
ERα gene *Xba*I polymorphism	−0.117	0.051	−0.263	0.121	−0.327^*^	0.037	−0.007	0.959	−0.124	0.214	−0.084	0.564

^*^ *p* < 0.05.

ERα, estrogen receptor α.

### Effect of low BMD risk factors and ERα gene *Xba*I polymorphism on BMD

In multiple regression analyses, sports types (*β* = −0.421; *p* = 0.000), BMI (*β* = −0.276; *p* = 0.000), age at menarche (*β* = −0.119; *p* = 0.016), and *Xba*I polymorphism (*β* = −0.128; *p* = 0.009) remained robust independent predictors of BMD. However, prior stress fractures (*β* = −0.076; *p* = 0.120) and menstrual cycles (*β* = −0.001; *p* = 0.982) were excluded.

In the XX+Xx genotype, sports types (*β* = −0.434; *p* = 0.000) and BMI (*β* = −0.308; *p* = 0.001) were significantly associated with BMD, but age at menarche (*β* = −0.067; *p* = 0.483), prior stress fractures (*β* = −0.015; *p* = 0.867), and menstrual cycles (*β* = −0.017; *p* = 0.853) were excluded. On the contrary, in the xx type, sports types (*β* = −0.436; *p* = 0.000), BMI (*β* = −0.275; *p* = 0.000), and age at menarche (*β* = −0.146; *p* = 0.014) were significantly associated with BMD, but prior stress fractures (*β* = −0.093; *p* = 0.122) and menstrual cycles (*β* = −0.002; *p* = 0.977) were excluded (Table 5).

**Table 5.** Influence of Low Bone Mineral Density Risk Factors and Estrogen Receptor α Gene *Xba*I Polymorphism on Total Body Bone Mineral Density

	All participants (*n* = 280)		XX+Xx genotype (*n* = 85)		xx genotype (*n* = 195)	
	β	*p*	β	*p*	β	*p*
Total body BMD						
Sports types	−0.421^*^	0.000	−0.434^*^	0.000	−0.436^*^	0.000
BMI	−0.276^*^	0.000	−0.308^*^	0.001	−0.275^*^	0.000
Age at menarche	−0.119^*^	0.016	−0.067	0.483	−0.146^*^	0.014
Prior stress fractures	−0.076	0.120	−0.015	0.867	−0.093	0.122
Menstrual cycles	−0.001	0.982	0.017	0.853	−0.002	0.977
ERα gene *Xba*I polymorphism	−0.128^*^	0.009				

^*^ *p* < 0.05.

XX, homozygous dominant; Xx, heterozygous; xx, homozygous recessive.

## Discussion

In this study, we investigated the effect of low BMD risk factors and ERα gene polymorphisms on BMD and whether the sensitivity of low BMD risk factors to BMD depends on ERα gene polymorphisms in collegiate female athletes.

About the relationship between low BMD risk factors and BMD, no significant correlation was observed in the endurance type, but significant correlation was observed between age at menarche and BMD in the aquatic type, BMI and BMD in the ball type, and prior stress fractures and BMD in the high-load type.

In the endurance type, as a factor that did not show the relationship between low BMD risk factors and BMD, it is considered that excessive training to improve competition performance exhibits negative influence on BMD, because previous studies have reported the effect of participation in endurance-type sports (*e.g.*, cross-country or long distance) on reduced BMD,^4,37^ and in the current study, endurance-type sports have shown the lowest BMD value. In the high-load type, significant correlation was observed between prior stress fractures and BMD. The ACSM consensus statement indicated that prior stress fractures were a high or moderate risk of the triad.^2^ In contrast, previous studies reported that sports participation with large mechanical stress may improve BMD.^4,7^ Therefore, as mechanical stress to the bone could not be achieved due to inactivity resulting from stress fractures, athletes of the high-load type with prior stress fractures may manifest a low BMD. Conversely, it is considered that high-load type sports and a relatively low BMD tended to cause stress fractures. As regards the significant correlation observed between age at menarche and BMD in the aquatic type, and BMI and BMD in the ball type, it is difficult to guess that no previous study has been reported. However, based on the results in the current study, at least the effect of low BMD risk factors on BMD may differ between each sports type. Therefore, further studies should investigate the effect of low BMD risk factors on BMD in each sports type.

In this study, multiple regression analysis showed that sports type, BMI, age at menarche, and *Xba*I polymorphism remained robust independent predictors of the total body BMD. Tenforde et al. investigated the effect of the triad risk factors such as BMI, menarche, menstrual abnormality, prior stress fractures, prior eating disorders, and participation in sports types on BMD by using multiple regression analyses. Their result suggested that athletes in low-impact and non-impact sports (*e.g.*, cross-country and swimming, respectively) and athletes with low BMI and oligomenorrhea/amenorrhea are at the highest risk for reduced BMD.^4^ Gibbs et al. evaluated the association between the triad risk factors and low BMD in exercising women using multivariable analyses. Their analyses revealed that delayed menarche, lean sports/activity participation, and lean body mass were the strongest predictors of low BMD when adjusting for BMI status, menstrual status, dietary restraint status, and age. In addition, in the combined risk factors, participants with delayed menarche+low BMI, delayed menarche+low body weight, and delayed menarche+elevated dietary restraint demonstrated the highest percentage of low BMD, suggesting a dose-response association between the number of the triad risk factors and BMD in exercising women.^9^

Although the results of the current study were consistent with those of previous studies, prior stress fractures and menstrual cycles were excluded in the present multiple regression analysis. As a factor that did not show the association between prior stress fractures and BMD, it is considered that stress fracture occurs not only with low BMD but also with differences in competition characteristics, because prior stress fractures occurred in 20% of participants in the high-load type, whereas prior stress fractures occurred in 19% in all study participants. As a factor that did not show association between menstrual cycles and BMD, menstrual cycles for 1 year were used for the analysis. In the current study, menstrual abnormality such as amenorrhea was set as a low-BMD risk factor, because it is associated with estrogen deficiency, which increases bone resorption and BMD reduction.^1,2^ However, as the period from menarche to the start of this experiment in participants was 6.4 years on average and some participants manifested high BMD such as those with menstrual abnormality because of the high-load-type sports, menstrual cycles in the previous year might not be an adequate indicator for assessing its effect on BMD in the study participants.

This study demonstrated that the xx genotype of *Xba*I polymorphism was associated with low BMD. Thus, *Xba*I polymorphism of the ERα gene as an independent predictor of multiple regression analysis was related to BMD; however, *Pvu*II polymorphism was not related to BMD. Previous studies regarding the association between ERα gene polymorphisms and BMD mainly targeted postmenopausal women, and results were significantly associated with BMD^25–28^ or were not associated with BMD.^29–32^ In addition, previous studies reported that the xx genotype of *Xba*I polymorphism was associated with low BMD^28^; in contrast, the XX genotype was associated with low BMD.^27^ Similarly, the pp genotype of *Pvu*II polymorphism was reported to be associated with low BMD,^25^ or the PP genotype was associated with low BMD.^27^ Therefore, results are conflicting. Gennari et al. suggested various factors that would account for the contradictory findings, including genotyping error, ethnic or environmental differences among populations, differences in age and menopausal status, and study design.^33^ To closely examine these conflicting results, Ioannidis et al. have evaluated the effect of ERα gene *Pvu*II and *Xba*I polymorphism on BMD by using meta-analysis. As a result, although *Pvu*II polymorphism was not associated with BMD, individuals with the xx type and Xx type of *Xba*I polymorphism had lower BMD values than those with the XX type.^38^ Thus, the results of the current study correspond with the result of Ioannidis et al.

Estrogens exert beneficial effects on the development and maintenance of the skeleton, and skeletal effects of estrogen are mediated by its binding to specific ER localized at the cytosol and intranucleus.^19^ It is clear that ERα gene plays an important role in bone homeostasis because of the occurrence of osteoporosis in men who have nonsense mutation of the ERα gene,^39^ or the BMD in the ERα gene knockout mouse was 20%–25% lower than that in the wildtype mouse.^40^ However, mechanisms about the effect of ERα gene polymorphisms on BMD are still unclear. Regarding these mechanisms, Kobayashi et al. hypothesized that these polymorphisms may be linked with exon mutation and contribute to changing the ER protein function or may be linked with mutations of other intron or regulatory elements and affect the levels of expression through transcriptional regulation.^24^

To investigate whether the sensitivity of low BMD risk factors to BMD depends on ERα gene *Xba*I polymorphism, we conducted multiple regression analyses in each *Xba*I polymorphism (*i.e.*, XX+Xx genotype and xx genotype, respectively). The results showed that sports types and BMI were significantly associated with BMD in the XX+Xx genotype. On the contrary, sports types, BMI, and age at menarche were significantly associated with BMD in the xx genotype. The sensitivity that sports types and BMI effect on bone response might not be different between *Xba*I polymorphism because these factors were associated with BMD for both XX+Xx genotype and xx genotype.

Kondo et al. examined the influence of ERα gene polymorphisms on BMD in response to habitual exercise in Japanese women and reported that women carrying the xx genotype may have increased BMD due to frequent exercise, but not those carrying the XX and Xx genotypes.^34^ In our study, the association between competition type and BMD for both XX+Xx genotype and xx genotype was recognized. Thus, our result was different from that in a previous study. Factors contributing to this difference were considered variations in study design, including differences in the heterogeneity of the participants or exercise load. As increasing BMI means weight gain relative to height, increased BMD is associated with increased load per bone area. Overt signs of low energy availability can be indicated by low energy stores such as low BMI,^2^ which may have affected BMD reduction because of the failure to supplement sufficient nutrition for bone formation. Thus, these may be factors that are strongly associated with BMD without being affected by genetic polymorphisms.

On the contrary, the effect of age at menarche on BMD was different in *Xba*I polymorphism, and athletes carrying the xx genotype were associated with delayed menarche and low BMD, but athletes carrying the XX+Xx genotype were not. As mentioned earlier, ERα gene plays an important role in bone homeostasis; however, mechanisms that affect ERα gene polymorphisms on BMD remain unclear. In our study, one of the factors associated between delayed menarche and low BMD in the xx genotype is decreased bone formation or increased bone resorption with low estrogen state, whereas XX or Xx genotype may have resistance to low BMD risk, such as low estrogen state.

### Limitations

The results of this study suggested that the effect of low BMD risk factors on BMD depends on sports types; thus, further study should investigate the effect of low BMD risk factors on BMD in each competitive sport. Moreover, low energy availability, which is one of the triad, was not sufficiently assessed because we did not conduct research on nutrition intake. Further study should investigate the effect of nutrition state on BMD between genetic polymorphisms. In this study, for multiple regression analysis, with each assessment item as an independent variable and BMD as a dependent variable, we used Japanese mean values such as 22 kg/m^2^ for BMI and 12.2 years for age at menarche as cutoff values. This is to analyze various athletes such as those of the high-load and endurance types included in this study. However, with the wide range of sports types, further study should consider the cutoff value that suits each sport. We focused on the ERα gene polymorphisms as bone metabolism-related gene polymorphisms, but other bone metabolism-related gene polymorphisms such as VDR gene and aromatase gene may be associated with BMD in female athletes. Therefore, if research using combination of multiple genetic polymorphisms was conducted, a more detailed relationship between low BMD risk factors and bone response will be revealed. In the future, to classify multiple genetic polymorphisms, a large research study for female athletes is necessary.

## Conclusion

In conclusion, to the best of our knowledge, this is the first study that investigated the association between low BMD risk factors and ERα gene polymorphisms and BMD. In Japanese collegiate female athletes, sports participation in endurance, esthetic, and aquatic types and low BMI are associated with low BMD. In addition, delayed menarche may negatively affect BMD in athletes carrying the xx type of ERα gene *Xba*I polymorphism.

## Abbreviations Used

ACSM: American College of Sports Medicine
BMD: bone mineral density
BMI: body mass index
ERα: estrogen receptor α
HWE: Hardy–Weinberg equilibrium
SD: standard deviation
VDR: vitamin D receptor
